# Future challenges for intervention research in health and lifestyle research—A systematic meta-literature review

**DOI:** 10.3402/qhw.v10.27326

**Published:** 2015-08-14

**Authors:** Lars Kristén, Andreas Ivarsson, James Parker, Kristina Ziegert

**Affiliations:** School of Health and Welfare, Halmstad University, Halmstad, Sweden

**Keywords:** Health, lifestyle, meta-literature review, meta-synthesis

## Abstract

The overall aim of this systematic meta-literature review was to (1) summarize the findings of review studies focusing on health determinants, (2) give an overview of intervention studies that have been used to facilitate health and lifestyle, and (3) provide recommendations for future studies in health promotion. A literature review, using a meta-method, was conducted to identify health and lifestyle research based on research articles related to health changes. The search yielded a total of 561 unique citations and finally 24 citations remained. Of those, 11 studies focused on health determinants, whereas 13 focused on interventions for health promotion. Results from this meta-synthesis led to four recommendations for the design of future intervention studies. (1) To increase the likelihood of capturing different biopsychosocial aspects of health, researchers from different scientific disciplines should collaborate in the design, implementation, and evaluation of the study. (2) It is recommended to use theoretical frameworks that focus on health determinants in longitudinal studies with a repeated measures design. (3) Studies should involve behavioral interventions. (4) Design face-to-face intervention studies where the participant can interact with other persons.

Health and well-being are two concepts that are widely discussed within today's society. One of the major challenges concerning these concepts has been to find widely accepted definitions (Dodge, Daly, Huyton, & Sanders, 2012). There is currently no one definition that has gained full acceptance by society. This has led to the research community using several different broad and unspecific definitions (Forgeard, Jayawickreme, Kern, & Seligman, 2011). The concept of health is often perceived to originate from medical science (Hallberg, 2010). The World Health Organization (WHO, 1947, p. 100) defines health as “a state of complete physical, mental and social well-being and not merely the absence of disease or infirmity.” This definition is not free from controversy, in particular due to the use of the word “complete” which implies absolute physical, mental, and social well-being as one of the fundamental rights of every human being, a perhaps somewhat purist view of health. Consequently, approximately 30 years ago, the Ottawa Charter for Health Promotion (1986) proclaimed that to reach the state of complete health, the group or individual should have the resources necessary to realize aspirations and cope with an ever changing environment. Therefore, health is seen as a “resource for everyday life” rather than as an overarching goal of human life. Furthermore, the charter proclaimed health as a positive concept emphasizing social and personal resources that are not just the responsibility of the health sector, but go beyond healthy lifestyles to well-being.

In the Jakarta declaration (1997), WHO addressed the challenges facing health promotion in the twenty-first century, including trends such as urbanization, increased numbers of older people, increase in number of chronic diseases, greater recognition of mental health problems, health as a human right that helps people to lead socially and economically productive lives, and above all, poverty as the greatest danger to health. Given that health is an important factor from a societal perspective, there is a need for more knowledge-based work in the welfare and healthcare sector (National Board of Health and Welfare, 2009; Schantz, 2012; Swedish National Institute of Public Health, 2008). Of special importance is to design and conduct health promotion programs that could address the challenges faced by the population of today. The Swedish welfare state is based on justice and equality, as preventing disparities in health and living conditions is an important social task. Therefore, a society and community task is to prevent disparities in health and living conditions between groups living in and contributing to the society (National Board of Health and Welfare, 2009). Health challenges faced by Europe and the Western world include the increased support and care needs of an aging population, the social marginalization of disabled people, as well as preventing lifestyle-related problems such as obesity and mental illness. These are examples of challenges where the knowledge of conditions, context, and change within society need to be developed. These challenges have been emphasized by the World Health Report 2013 (WHO, 2013). In line with these challenges, the United Nations has developed three “Millennium Development Goals,” regarding maternal health, child health, and control of communicable diseases, which are guidelines for all countries working with health promotion. Furthermore, the European Union “Horizon 2020” (European Commission, 2014) highlights health demographic change and well-being as an important societal challenge; more specifically, it aims “to keep older people active and independent for longer” and supports the development of new, safer, and more effective interventions. There is a need to design and conduct health promotion programs that can match the challenges observed in today's society.

A major perspective in health and lifestyle research is to investigate what determinants are associated with health. A large body of studies focusing on determinants of health and well-being have been conducted during the last decades (e.g., Glasgow, Klesges, Dzewaltowski, Bull, & Estabrooks, 2004). Health and lifestyle, and, in particular, physical activity research among children and youth with disabilities takes into account both barriers and facilitators connected with the perceptions related to personal, social, environmental, and policy or program factors. The complex and multifactored context in a society for this group leads to a higher grade of non-participation in physical activity (Shields, Synnot, & Barr, 2012). Also, parents of children with disabilities report lacks in health and well-being such as stress-related problems, depressive symptoms, and social challenges (Hallberg, 2014). Furthermore, Kahn et al. (2002) present a logical framework that suggests that health determinants can be divided into three different categories: environmental and policy determinants (e.g., facilities), behavioral and social determinants (e.g., behavioral management skills, social support), and information-based determinants (e.g., providing information). How humans behave is closely related to health conditions. Global disease is increasingly related to lifestyle conditions, and patient-centered health educational strategies have been developed in educational settings over the last decades; however, it could be questioned whether educational strategies and learning have much improved in both medical and educational institutions and contexts (Alexander et al., 2012).

Adequate interventions are an important tool for initiation of improved health behavior; however, maintenance has proven to be elusive and requires separate processes and skills (Voils et al., 2014). In relation to the design of health promotion intervention studies, several recommendations have been addressed. First, the intervention is best designed using relevant theories (Glanz & Bishop, 2010). Theories should be used to understand and explain barriers for health behaviors and how to design interventions to overcome these barriers (Hochbaum, Sorenson, & Lorig, 1992). One category of theories that has been suggested to give essential contribution to such interventions is health psychological theories (Kok, Schaalma, Ruiter, Empelen, & Brug, 2004). Three of the most frequently used theories are the “health belief model,” “social cognitive theory,” and “trans theoretical model of change” (Glanz & Bishop, 2010). Even if the theories are focused on different variables, they share the underlying dimension that they aim to predict and understand behaviors (Fishbein & Yzer, 2003; Glanz & Bishop, 2010).

When it comes to the delivery of health interventions, several different approaches have been suggested. Two of the most common approaches are face-to-face and online computer-tailored interventions (Soetens, Vandelanotte, Vries, & Mummery, 2014). For example, it is possible to promote lifestyle changes and improve functioning for adults by using motivational interviewing (Cummings, Cooper, & Cassie, 2009). Computer-tailored interventions are suggested as one starting point for facilitating improvement in behaviors related to chronic disease and health promotion (Krebs, Prochaska, & Rossi, 2010). Examples of peer-based interventions, which have shown progressive results attempting to affect health-related behavior changes, are behavior changes in physical activity, smoking, and condom use (Webel, Okonsky, Trompeta, & Holzemer, 2010).

Most health promotion studies have been evaluated from a positivistic perspective using quantitative measurements and statistical analysis. Recently, more and more studies have used qualitative evaluation procedures for example, in the implementation process of public health programs. This shift in evaluation procedures was to take into account the stakeholders’ perspective and provide results useful for the design and planning of forthcoming programs to be able to evaluate the effectiveness of these strategies from the stakeholder's point of view (Tayabas, León, & Espino, 2014). Another aspect that is discussed in relation to evaluation of health promotion programs is the need for evaluating mediation factors, as many intervention studies are designed to change a number of different variables (e.g., motivation) that will lead to behavioral changes because of changes in mediation mechanisms (Di Noia & Prochaska, 2010). To, therefore, gain knowledge of why an intervention is or is not effective, it is important to also evaluate mediational factors. By doing that, it is possible to revise the intervention to better target those mediators (Reynolds, Yaroch, Franklin, & Maloy, 2002).

A gap between research and practice exists, indicating increasing knowledge on lifestyle-related conditions in both academic and professional settings (Burniston, Eftekhari, Hrabi, Worsley, & Dean, 2012). More specifically, several weaknesses in methods, study design, and characteristics in relation to previous interventions have been addressed. Therefore, the lifestyle behavior change interventions seem to need an agenda for better decision making, for example, that essential study details should be better reported, and evaluators should use good practice guidelines (Alayli-Goebbels et al., 2013). Another aspect concerning evaluation of intervention studies is to take both the context and the group of participants into consideration when interpreting the results (Glasgow et al., 2004).

To overcome the shortcomings addressed in previous health promotion studies, there is a need to evaluate the effectiveness of previous studies. By summarizing the results from previous studies, it would be possible to provide recommendations for the design and evaluation of health intervention studies.

The present study is part of a project headed by the Center of Research on Welfare, Health and Sport contributing to future challenges in research on health in groups and individuals with the perspective related to research combining caring, disability studies, and sports psychology. The overall aim of this systematic meta-literature review consisting of systematic reviews published from 2004 to 2014 is to provide insights into factors that influence health and lifestyle in different populations. The aim was to (1) summarize the findings of review studies focusing on health determinants, (2) give an overview of intervention studies that have been used to facilitate health and lifestyle, and (3) provide recommendations for future studies in health promotion.

## Methods

The meta-synthesis was chosen for our synthesis of research studies using a health and lifestyle review format and meta-questions analysis. The method determines how the meta-synthesis methods have been interpreted by the researchers, and explores accuracy and structure (Paterson, Thorne, Canam, & Jilings, 2001). A meta-literature review approach includes various methods that aim at developing both new knowledge based on critical analysis, as well as to arrive at new insight beyond the original focus of research (Bondas & Hall, 2007). The process of our meta-synthesis included the following five phases:

1. Literature search for articles.
2. Selection of relevant articles after repeated reading and appraisal of the articles.
3. Studies were considered if they focused on meta-literature review of health determinants and interventions for health promotion.
4. Information was extracted from each admissible study on: researcher, year, country, research focus, health definitions, and results.
5. The studies included in this meta-synthesis have shown that the concept of health and well-being can include aspects from different areas.

## Literature search for articles

A literature search was conducted to identify health and lifestyle research based on the research articles related to health changes and meta-method study. For the literature review, the following databases were searched: ABI Inform (2004–October Week 43 2014); PubMed (2004–October Week 43 2014); CINAHL (2004–October Week 43 2014); PsycINFO (2004–October Week 43 2014); SportDiscus (2004–October Week 43 2014); ERIC (2004–October Week 43 2014); Sociological abstracts (2004–October Week 43 2014). The following keywords and combinations of keywords were used in and modified for other databases where appropriate: health*OR “life style” Or meta-framework OR meta-re* OR meta-synthesis OR meta-theory OR meta-method. Limits applied: peer-reviewed, article on English, not related to illness.

## Selection of relevant articles after repeated reading and appraisal of the articles

**Figure 1:**
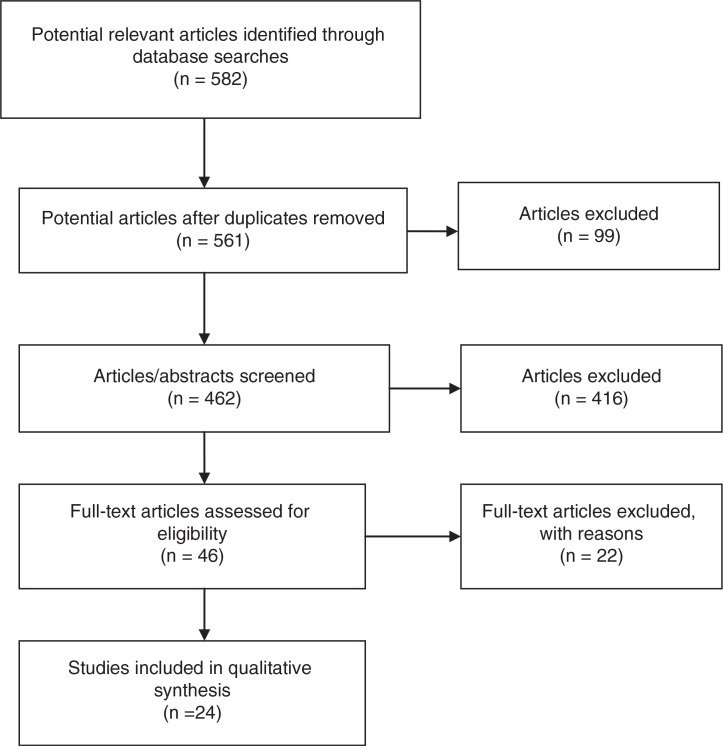
Description of the selection process of the studies.

The search yielded a total of 561 unique citations. The research team was divided into three groups and each group reviewed approximately one-third of the citations. After the citation titles were read, 99 citations were excluded by title. In the next step, the abstracts were scrutinized and 461 citations were excluded. The reasons for exclusion, at this stage, were: the studies had no meta-review or summaries of meta-analyses, or the outcome variables were considered to be related to illness. The remaining 46 citations were read in full, and a further 22 citations were excluded. At this stage, the reason for exclusion was that the outcome variables were considered to be related to illness. Finally, 24 citations remained (for the full flow chart, see Figure 1). Reasons for exclusion of the article were: comparison between predictions and interventions related to health and lifestyle, and any non-scientific articles.

## Findings

### Meta-synthesis in different research areas

In total, 24 studies were included in our meta-synthesis. Of those, 11 studies focused on health determinants, whereas 13 focused on interventions for health promotion.

## Studies focusing on health determinants

The studies focusing on health determinants/predictors were published between 2004 and 2014. Of the reviews selected for our meta-synthesis, 91% presented their selection process of studies. In these reviews, a total of 483 studies were included (for more information about the studies, see Table I).

**Table I:** Overview of health determinant studies included in the systematic meta-literature review.

Researcher (s), year, country	Research focus	Method, sample, and years	Health definitions	Results
Adams et al., 2011, USA	Social and leisure activities association with well-being	Systematic review, 42, 1995–2009	Subjective well-being, metal health	Most studies showed a positive association between actively participation in social and leisure activities and psychosocial well-being
Bailey, 2006, England	Physical education and sport in schools impact on health	Systematic review, NA, NA	Physical development, lifestyle development, affective development, social development, cognitive development	Physical education and sport in schools were related to a healthier lifestyle
Furnée et al., 2008, The Netherlands	Health educations association with self-reported health	Meta-analysis, 40, NA	Self-reported health	There was a positive association between health education and self-reported health
Kyröläinen et al., 2010, Finland	Relationship between physical fitness, obesity, and health	Systematic review, 85, 1966–2009	Obesity and physiological health	The result showed that the participants’ lifestyle and health behaviors were closely related. More specifically, high volume physical activity and good physical fitness are associated with enhanced health
Lubans et al., 2010, Australia	The mastery of fundamental movement skills (FMS) relationship with potential health benefits in children	Systematic review, 21, to June 2009	Psychological, physiological and behavioral health outcomes	The study reported positive relationships between FMS and physical activity as well as cardio-respiratory fitness. A negative relationship between FMS and weight status was also reported in the study
Shields et al., 2012, Australia	Barriers and facilitators for participation in physical activity among children with disability	Systematic review, 14,to September 2010	Physical activity behavior	Barriers for physical activity participation were lack of knowledge and skills, fear, parental behavior, inadequate facilities, programs, and staff capacity Facilitators were child's desire to be active, practicing skills, involvement of peers, family support, accessible facilities, skilled staff, and information
Södergren, 2013, Sweden	Predictors for a healthy lifestyle	Systematic review, 20, 1998–2012	Physical activity behavior, smoking, alcohol intake, and fat intake	A healthy lifestyle facilitated good health in late life
Tammelin, 2005, Finland	Predictors of adulthood physical activity	Systematic review, 32, NA	Physical activity behavior	High levels of physical activity, participation in organized sports, good cardio-respiratory fitness, and high marks in physical education at school were the strongest predictors of physical activity participation in adulthood
Trudeau and Shephard, 2005, Canada	School physical education programs effects on the level of and attitudes to physical activities in children and adults	Systematic review, 132, 1970–2003	Physical activity behavior	High quality school PE programs increased the physical activity level in children. Moreover, high quality PE programs increased the likelihood of a positive attitude towards physical activities in adulthood
Van der Horst et al., 2007, The Netherlands	Correlates of physical activity and sedentariness in children	Systematic review, 60, 1999–2005	Physical activity behavior	Self-efficacy, parental physical activity, and parental support were positively associated with participation in physical activity. Socioeconomic status and parent education were inversely associated with adolescent sedentary behaviors
Yarcheski et al., 2004, USA	Predictors of positive health practices	Meta-analysis, 37, 1983–2003	Participation in health promotion activities (e.g., exercise and relaxation)	Loneliness, social support, perceived health status, self-efficacy, future time perspective, self-esteem, hope, and depression all had moderate effects on positive health practices

NA, not available.

The findings from the review showed that there are a number of different factors that could be discussed as health determinants. For example, five review papers summarized research on predictors for a physically active lifestyle. More specifically, Tammelin (2005) found that high levels of physical activity in childhood, together with participation in organized sport activities, good cardio-respiratory fitness, and high grades in physical education were all predictors of a physically active lifestyle in adulthood. In line with this result, Trudeau and Shephard (2005) reported that high quality school programs for physical education increased the likelihood for a physically active lifestyle in children as well as a more positive attitude towards physical activities in adulthood. Another review that also aimed to investigate determinants for a physically active lifestyle among children found that a high level of self-efficacy and high levels of parental support, as well as the parents having a physically active lifestyle, all increased the likelihood of a physically active lifestyle for the child (Van der Horst, Paw, Twisk, & Van Mechelen, 2007). One more determinant of a physically active lifestyle was to be able to master fundamental movement skills (Lubans, Morgan, Cliff, Barnett, & Okely, 2010). The fifth reviews focused on predictors of a physically active lifestyle and included studies that had investigated barriers and facilitators for a physically active lifestyle among children with disabilities (Shields et al., 2012). The result showed that facilitators for a physically active lifestyle were a desire to be active, practicing skills, involvement of peers, family support, accessible facilities, information, and skilled staff.

In the above-mentioned reviews, a physically active lifestyle is considered as a health outcome. Other reviews have found a physically active lifestyle as a health determinant. For example, several of the reviews showed that a physically active lifestyle was associated with enhanced health status both in children (Bailey, 2006) and adults (Kyröläinen, Santtila, Nindl, & Vasankari, 2010; Södergren, 2013). There are also studies that have focused on other health determinants/predictors than a physically active lifestyle. Examples of health determinants were high levels of social support, self-efficacy, self-esteem, and hope (Yarcheski, Mahon, Yarcheski, & Cannella, 2004), participation in social and leisure activities (Adams, Leibbrandt, & Moon, 2011), as well as participation in health education (Furnée, Groot, & Van den Brink, 2008).

To sum up, most studies have collected information about health determinants in children and/or youth populations. Physical activity is discussed as both a health determinant and a health outcome. In addition, psychosocial variables, such as social support, self-efficacy, and hope were identified as health determinants. Moreover, participation in health education as well as social activities was also found to be associated with health and well-being.

## Studies focusing on interventions for facilitating health and well-being

The studies focusing on interventions for health promotion were published between 2004 and 2014. Of these, 85% presented the selection process of studies included in their reviews. In these reviews, a total of 719 studies were included (for a summary of the studies, see Table II).

**Table II:** Overview of intervention studies included in the systematic meta-literature review.

Researcher (s), year, country	Research focus	Method, sample, and years	Health definitions	Results
Camero et al., 2012, USA	Physical activity interventions effect on mental health determinants in children	Systematic review, 8, 2000–2011	Psychological health (e.g., depression, global self-worth, self-efficacy)	Physical activity prevented depression and increased global self-worth and self-efficacy
Conn et al., 2011, USA	The effectiveness of interventions aimed to increase physical activity	Meta-analysis, 358, 1960–2007	Physical activity	The overall result showed that the interventions were effective in promoting physical activity (ES=0.19). Behavioral interventions were more effective than cognitive interventions. Face-to-face delivery was more effective than interventions with contact via phone or mail
Dale et al., 2014, England	Healthy lifestyle interventions influence on mental health and well-being	Systematic review, 29, to April 2013	Physical health, psychological health (e.g., depression, anxiety, mental function, quality of life)	Most studies (*n*=25) showed improvements on mental health and well-being
Garrett et al., 2011, New Zealand	Physical activity interventions impact on costs in primary care	Systematic review, 13, 2002–2009	Physical activity behavior	Most interventions to increase physical activity were cost-effective
Kaspin, Gorman, and Miller, 2013, USA	Employer-sponsored wellness programs effect on health	Systematic review, 20, 2005–2011	Changes in health risk factors, practice of healthy behaviors (e.g., exercise and stress management), and patient-reported outcomes (e.g., quality of life)	Employer-sponsored wellness programs improved health among the employees
Kruger et al., 2009, USA	Physical activities association with cognitive health	Systematic review, 160, 1980–2005	Cognitive health	No strong association between physical activities and cognitive health. Nevertheless, a positive association was found in studies that had moderate intensity physical activity
Kuoppala et al., 2008, Finland	Work related health promotion activities influence on job well-being and work ability	Systematic review and meta-analysis, 46, 1970–2005	Work health, work ability, and mental health	Work health promotion activities decreased sickness absence (RR=0.78) and increased work-abilities (RR=1.38). Moreover work health promotion activities (RR=1.39) as well as exercise (RR=1.25) increased mental well-being
Muller-Riemenschneider et al., 2009, Germany	Cost-effectiveness of interventions promoting physical activity	Systematic review, 8, 1998–2008	Physical activity behavior	Behavioral interventions increased physical activity level. Environmental interventions were more cost-effective (800 euros/year for the behavioral intervention)
Plotnikoff and Karunamuni, 2011, Canada/Australia	Physical activity promotion	Systematic review, NA, 2009–2011	Physical activity behavior	Social-cognitive theories could be useful when designing health intervention studies. During the life course, a number of different physical activities can be implemented through recreational, transportation, and occupational activities
Rew, Johnson, Henkins, and Torres, 2004, USA	Holistic nursing interventions to improve adolescent health	Systematic review, NA, NA	Use of drugs, physical activity, nutrition, sexual behavior, and violence	Participation in physical activities had positive effects on physical health
Rhodes, Warburton, and Murray, 2009, USA/Canada	Characteristics of physical activity guidelines and their effect on adherence to prescribed physical activity	Systematic review, 27, to November 2007	Physical activity behavior	The type of the physical activity had a weak relationship to physical activity behavior
Rongen, Robroek, Lenthe, and Burdorf, 2013, The Netherlands	The effectiveness of workplace health promotion	Meta-analysis, 18 (21 interventions), up to June 2012	Self-reported health, work absence due to sickness, work productivity, and work ability	The result showed that workplace health promotion interventions had positive influence on health outcomes
Van Sluijs et al., 2004, The Netherlands	The effects of stage-based lifestyle interventions in primary care	Systematic review, 29, to July 2002	Smoking behaviors, physical activity, and fat intake	Weak evidence for stage-based lifestyle interventions effect on: physical activity, smoking behavior. Strong evidence for interventions effect on fat intake

NA, not available.

Of the studies that met the inclusion criteria, six aimed at evaluating research that had investigated the effectiveness of interventions targeting promotion of physical activity. Muller-Riemenschneider, Reinhold, and Willich (2009) found that interventions based on behavioral strategies were cost-effective when it came to promoting physical activities in healthy adults. Moreover, they concluded that environmental interventions could have the potential to be even more cost-effective, but more research is needed to investigate whether this is the case. In line with this, Garrett et al. (2011) reviewed the costs for physical activity interventions where they showed that most physical activity interventions were cost-effective. More specifically, they concluded that the costs for most physical activity interventions were comparable with pharmaceutical interventions. Among the included studies that investigated different interventions effectiveness on physical activity behavior, Conn, Hafdahl, and Mehr (2011) found, in their meta-analysis, that behavioral interventions (e.g., goal-setting, physical activity feedback, self-monitoring) were more effective than other intervention strategies (e.g., cognitive). Another finding reported by the authors was that interventions in which participants had the opportunity to have face-to-face delivery of the interventions were more effective than interventions based on other delivery forms (e.g., mail, phone, online). This result was supported by another study that evaluated physical activity guidelines interventions, such as recommendations of daily activities and impact on physical activity behavior (Rhodes, Warburton, & Murray, 2009). The results showed weak effects of these cognitive-based intervention strategies on physical activity behavior. Similar results were reported by Van Sluijs, Van Poppel, and Van Mechelen (2004) that reported weak effects of stage-based lifestyle interventions, such as physical activity counseling, stage-oriented material, and motivational interviewing on physical activity behavior. The sixth study included in our meta-synthesis emphasized, in contrast to the other reviews, the importance of applying theoretical-driven approaches from, for example, the social-cognitive paradigm (e.g., theory of planned behavior) when designing interventions to promote physical activity behavior. The authors also conclude that it is important to adjust the intervention to the target population because research has shown that, for example, people of different age groups respond differently to different models of delivery (Plotnikoff & Karunamuni, 2011).

Three of the included intervention reviews evaluated the impact of physical activity interventions on health. In one of the reviews, the result showed that the physical activity interventions reduced the risk for depression and increased psychological health (e.g., self-worth and self-confidence) among children and adolescents (Camero, Hobbs, Stringer, Branscum, & Taylor, 2012). Positive health effects of physical activity interventions were also found by Rew, Johnson, Henkins, and Torres (2004) who reported positive effects on physical health (cardiovascular fitness) in children. The two other reviews concluded that physical activity interventions had positive impact on health; one review found this relationship to be weak. More specific, Kruger, Buchner, and Prohaska (2009) reported a weak effect of physical activity interventions on cognitive health. Nevertheless, the same study also reported a strong effect of moderate intensity physical activity interventions on cognitive health.

In another study, the aim was to review health behavior change interventions’ effect on mental health and well-being (Dale, Brassington, & Kring, 2014). The results showed that health behavior change interventions, such as increased physical education activities in school, access to step-counters and behavioral/motivational counseling, had positive effects on mental health and well-being (e.g., decreased stress levels, increased quality of life).

Another context that review studies have focused on when it comes to health promotion is workplace interventions. Rongen, Robroek, Van Lenthe, and Burdorf (2013) reported that the effectiveness of workplace interventions (e.g., physical activities groups, seminars, web-based programs) had small effects on the employees’ health. In comparison, another review, with similar aim, showed that work health promotion programs had positive effects on mental well-being and work ability (Kuoppala, Lamminpää, & Husman, 2008). Similar results were reported by Kaspin, Gorman, and Miller (2013) that included studies using employer-sponsored wellness strategies into their review.

To sum up, most intervention studies have been effective in promoting health. Two of the reviews suggested that behavioral interventions were effective in promoting physically active behavior. Another finding was that face-to-face interventions could be more effective than interventions that delivered their messages in other forums. Studies including physical activity interventions reported, in general, improved health and well-being (e.g., psychological, physiological). Moreover, interventions that were performed at the workplace showed small-to-moderate effects on health and mental well-being.

## Discussion

The overall aim of this systematic meta-literature review consisting of systematic reviews published from 2004 to 2014 was to provide insights into factors that influence health and lifestyle in different populations. The aim was to (1) summarize the findings of review studies focusing on health determinants, (2) give an overview of intervention studies that have been used to facilitate health and lifestyle, and (3) provide recommendations for future studies in health promotion.

One important finding that might have an impact on both health prediction and health promotion research is that there are, within the included reviews, a number of different definitions of health (e.g., to be physically active, cognitive health, physical health, social health). According to the WHO (1947) definition, health is addressed as “a state of complete physical, mental and social wellbeing and absence from disease or infirmity.” In many of the included reviews, the health definitions are limited to just one of the aspects (e.g., physical health) in the WHO definition. This procedure might be discussed as a limitation because different health aspects might have different determinants, which might influence the conclusions from the different studies. Therefore, it is important to measure multiple health factors (i.e., psychological well-being, social well-being, physiological and metabolic health, physical capacity, and cognitive function) to be able to investigate and properly discuss the complex concept of health (Lara et al., 2013). It is, therefore, of importance to combine several different perspectives, such as psychological, sociological, nursing, and economical to improve the quality of health studies (Glanz & Bishop, 2010).

Concerning the reviews that focused on health determinants, most studies were performed on younger populations (children). In this group, one of the determinants was high level of parental support. One potential explanation for this link between health and parental support is that parents have been found to shape patterns of behaviors (e.g., health behaviors) in adolescents (De Bourdeaudhuij & Van Oost, 1998). More specifically, parents that are physically active and understand the importance of being physically active will probably encourage their children to perform health behaviors (e.g., be physically active, eat healthy food).

Another determinant was related to a physically active lifestyle, more specifically high quality school programs for physical education. Together with the findings in children from Bailey (2006), in adults from Kyröläinen et al. (2010), and Södergren (2013) regarding an enhanced health status along with a physically active lifestyle, this could be a starting point for a nationwide approach to daily physical activity in the whole society. In some European countries, the effort is to promote daily physical education in schools for all pupils with a visionary view of improving the public health in society. Physical activity among children and youth with disabilities takes in account both barriers and facilitators connected to the society and the individual needs of the group. A higher grade of non-participation in physical activity is shown compared with children and youth without disabilities (Shields et al., 2012). There seems to be a lack of knowledge about barriers and facilitators for a physically active lifestyle among children with disabilities, especially about how to adapt and motivate physical activity for this socially marginalized group in society.

Another possible factor that was found to facilitate health was participation in health education programs. This finding is in line with social cognitive theories that highlight knowledge about health risks and benefits of health behaviors, as a determinant of effective health practices (Bandura, 2004), which facilitates a healthy lifestyle. To develop knowledge about why it is important to have a healthy lifestyle as well as what activities could facilitate health will probably increase the participants’ motivation to have a healthy lifestyle. That the social context is important to facilitate health behaviors is also suggested in social cognitive theories. More specifically, factors such as social norms have been suggested to indirectly influence humans’ behavior through goal-setting (Bandura, 2004). Furthermore, Kuoppala et al. (2008) emphasized that leadership as well as working climate were two determinants of health and well-being. In this context, it is clear that the actions of other significant actors/persons will have great impact on the possibility of improving health through interventions.

One potential limitation with the studies conducted is that most of them used limited types of populations (e.g., children). It could, therefore, be difficult, based on the findings from such studies, to generalize the findings to other groups, times, and settings (Glasgow et al., 2004). This potential limitation could influence the possibility of designing effective interventions for other groups of participants. It could, therefore, be important to investigate whether similar health determinants are true for different groups of participants. To find health determinants that are similar for a range of populations will increase the likelihood of designing intervention studies that could be implemented on a society level.

Concerning intervention studies aimed at promoting health and well-being, several of the reviews suggest interventions focusing on behaviors (e.g., goal-setting, physical activity feedback, self-monitoring) to be more effective than other intervention strategies (e.g., cognitive). This finding supports the suggestion that health psychology theories such as trans-theoretical model of change and social cognitive theory, which focus on prediction of health behaviors (Fishbein & Yzer, 2003; Glanz & Bishop, 2010), are effective in using health promotion interventions as a base. One potential explanation for the effectiveness of behavioral interventions could be that humans participating in such interventions are targeting an actual behavior (Culter, 2004).

Another finding was that intervention studies based on face-to-face meetings seemed to be more effective than other types of interventions (e.g., mail contact). One potential reason for this is that a number of studies, within psychotherapy, have found that the most important predictor for the effectiveness of the intervention is the quality of the relationship between the therapist and the client (Cozolino & Santos, 2014). More specifically, the quality of the relationship will correlate with changes in the brain's network. How the brain's network is structured is associated with, for example, behavior (Cramer et al., 2011). To establish a good relationship with the participants in an intervention could, therefore, be one of the parameters that might increase the effectiveness of the intervention. To establish such a relationship is probably harder in other forums where the consultant and client do not meet.

Lastly, in all intervention studies, physical activity behaviors were included as an outcome of an intervention program. It is, therefore, speculated that physical activity behavior could be discussed as one mediator between health determinants and health outcomes. This suggestion is in line with the “logic framework” of Kahn et al. (2002) where physical activity behavior is suggested to mediate informational, behavioral and social, as well as environmental and policy determinants influence on health outcomes. It is, therefore, recommended that intervention studies should focus on programs that facilitate physical activity behavior.

Other reviews, included in this study, evaluated the cost–benefit of intervention studies. These studies showed that interventions based on behavioral strategies were cost-effective when it came to promoting physical activities in healthy adults (Muller-Riemenschneider, Reinhold, & Willich, 2009). Furthermore, environmental interventions could have the potential to be even more cost-effective, but more research is needed in this area. To design and evaluate such environmental interventions, the theoretical framework “determinants of health and well-being on our cities” from Barton and Grant (2013) could be used.

Finally, there seem to be conflict/uncertainty about the sampling of the meta-synthesis. There were strivings towards an ideal of a total sample as well as convenient or purposeful samples. The type of sample is seldom mentioned and would be expected in the description of the sampling criteria (Bondas & Hall, 2007). The studies included in the meta-synthesis were published in a variety of countries, with different analytical techniques of samplings, utilizing both convenience and purposive sampling and recruiting predictions and interventions in the health and lifestyle context.

## Conclusions and recommendations

Based on this systematic meta-literature review, a few recommendations for the design of future intervention studies are suggested. Our results indicate that there is clearly a need for research in health and particularly research using a comprehensive approach to health promotion.

Because the studies included in this meta-synthesis have shown that the concept of health and well-being can include aspects from different areas, such as biomechanics, physiology, pedagogy, psychology, and sociology, it is important to include these different areas in health research. One possibility to increase the likelihood of capturing different aspects of health is that researchers from different research perspectives (such as exercise/health psychology, caring, disability studies, and education) should collaborate in the design, implementation, and evaluation of the study. In such a group, it could be possible to capture the complex biopsychosocial phenomenon that is health.

Several of the included review studies indicated that intervention concepts targeting behaviors were effective in promoting health and well-being. Therefore, future studies are recommended to use theoretical frameworks focusing on prediction and understanding of behaviors. When discussing behaviors, it is also important to emphasize that research should evaluate both health outcomes and potential mediators in their evaluation of the studies. To be able to do so, it is essential to apply longitudinal designs with repeated measures (with minimum three measurement points). To do mediation analyses where the variables are measured at the same time is problematic because in such a design the processes between the variables are assumed to be instantaneous (Selig & Preacher, 2009).

We also conclude that because most of the studies included in the meta-review have focused on specific populations, it is recommended that future studies include several different populations in their designs. If an intervention program is effective within several populations, it could be a foundation for a community health promotion program targeting the majority of the population.

Finally, it is recommended to design intervention studies where the participant can interact with other persons who are willing to listen to his/her stories (Cozolino, 2010). This could, for example, be facilitated by face-to-face contact between a researcher and participant, or between the participants themselves.
